# Intensive Individualized Recovery in Chronic Post-stroke Disability: Functional Outcomes, Fatigue Modulation, and Implications for Extended Neuroplasticity

**DOI:** 10.7759/cureus.109230

**Published:** 2026-05-19

**Authors:** Nurkyz U Beishenalieva, Shafee U Rehman

**Affiliations:** 1 Exercise Physiology, Life University, Marietta, USA; 2 Faculty of Medicine, Ala-Too International University, Bishkek, KGZ

**Keywords:** chronic stroke, fatigue management, intensive functional recovery, neuroplasticity, stroke rehabilitation

## Abstract

Chronic post-stroke disability is commonly associated with limited recovery potential, often due to insufficient intensity and duration of conventional interventions. Emerging evidence suggests that neuroplasticity may persist beyond traditional recovery windows when interventions are appropriately intensified, individualized, and grounded in exercise physiology principles. This study aimed to evaluate functional recovery outcomes following an intensive, individualized recovery-oriented program in a patient with severe chronic post-stroke impairment and to explore the role of fatigue in modulating performance and adaptation. A single-case clinical study was conducted in a patient with long-standing post-stroke disability and minimal prior improvement. The intervention consisted of prolonged, high-dose daily recovery sessions incorporating task-specific training, neuromuscular re-education, endurance conditioning, and continuous real-time adaptation based on fatigue and performance. Clinical observations were supplemented with an illustrative dataset to model longitudinal trends and functional relationships. The patient demonstrated progressive improvements in mobility, balance, coordination, endurance, and independence in activities of daily living. Recovery tolerance and training capacity increased over time, enabling longer and more effective sessions. Longitudinal analysis indicated consistent functional gains and a negative association between fatigue and performance, suggesting that fatigue modulation may play a key role in optimizing recovery outcomes. This case supports the potential for meaningful functional recovery in chronic post-stroke populations through high-intensity, individualized, exercise physiology-based recovery strategies. The findings highlight the importance of intervention dose, adaptive programming, and fatigue management and suggest that recovery capacity may extend beyond conventional expectations. Further research using standardized outcome measures is warranted.

## Introduction

Stroke remains one of the leading causes of long-term disability worldwide and is frequently associated with persistent impairments in mobility, coordination, balance, endurance, and activities of daily living (ADL). Although significant neurological recovery commonly occurs during the acute and subacute phases, many patients continue to experience substantial functional limitations years after the initial cerebrovascular event. Chronic stroke-related disability often results in reduced independence, impaired quality of life, and decreased participation in social and physical activities [1]. Traditional rehabilitation approaches have historically assumed that recovery potential declines substantially after the early post-stroke period. However, emerging evidence suggests that neuroplastic adaptation may persist beyond conventional recovery windows when rehabilitation is delivered with sufficient intensity, repetition, and task specificity [2,3]. High-intensity and prolonged rehabilitation programs have therefore gained increasing attention as potential strategies to promote continued functional recovery in chronic stroke populations.

In addition to motor weakness and impaired neuromuscular control, post-stroke fatigue has emerged as a major but often underrecognized factor limiting rehabilitation participation and long-term functional progression. Unlike peripheral muscle weakness alone, post-stroke fatigue may involve multiple neurophysiological mechanisms, including altered central nervous system activation, autonomic dysfunction, inflammatory responses, impaired neuromuscular efficiency, and possible disturbances in cellular bioenergetics [4,5]. These factors may reduce exercise tolerance and endurance even in patients capable of performing basic motor activities. Despite increasing recognition of post-stroke fatigue, limited attention has been directed toward how fatigue tolerance influences participation in prolonged high-intensity rehabilitation programs, particularly in patients with severe chronic disability [6,7]. Many chronic stroke patients remain unable to tolerate extended rehabilitation sessions because of rapid exhaustion, limited endurance, and reduced recovery capacity. Consequently, rehabilitation intensity is often reduced, potentially limiting long-term functional improvement [8,9].

The present case is clinically notable because it describes a patient with severe chronic post-stroke disability and prominent fatigue-related rehabilitation limitation who underwent an intensive individualized rehabilitation program incorporating adaptive fatigue-management strategies, scheduled recovery periods, and prolonged task-specific training. The purpose of this case report is to describe the patient’s longitudinal functional progression during intensive rehabilitation and to explore the clinical relationship between fatigue tolerance, rehabilitation participation, and functional recovery in chronic stroke rehabilitation.

## Case presentation

**Figure 1:**
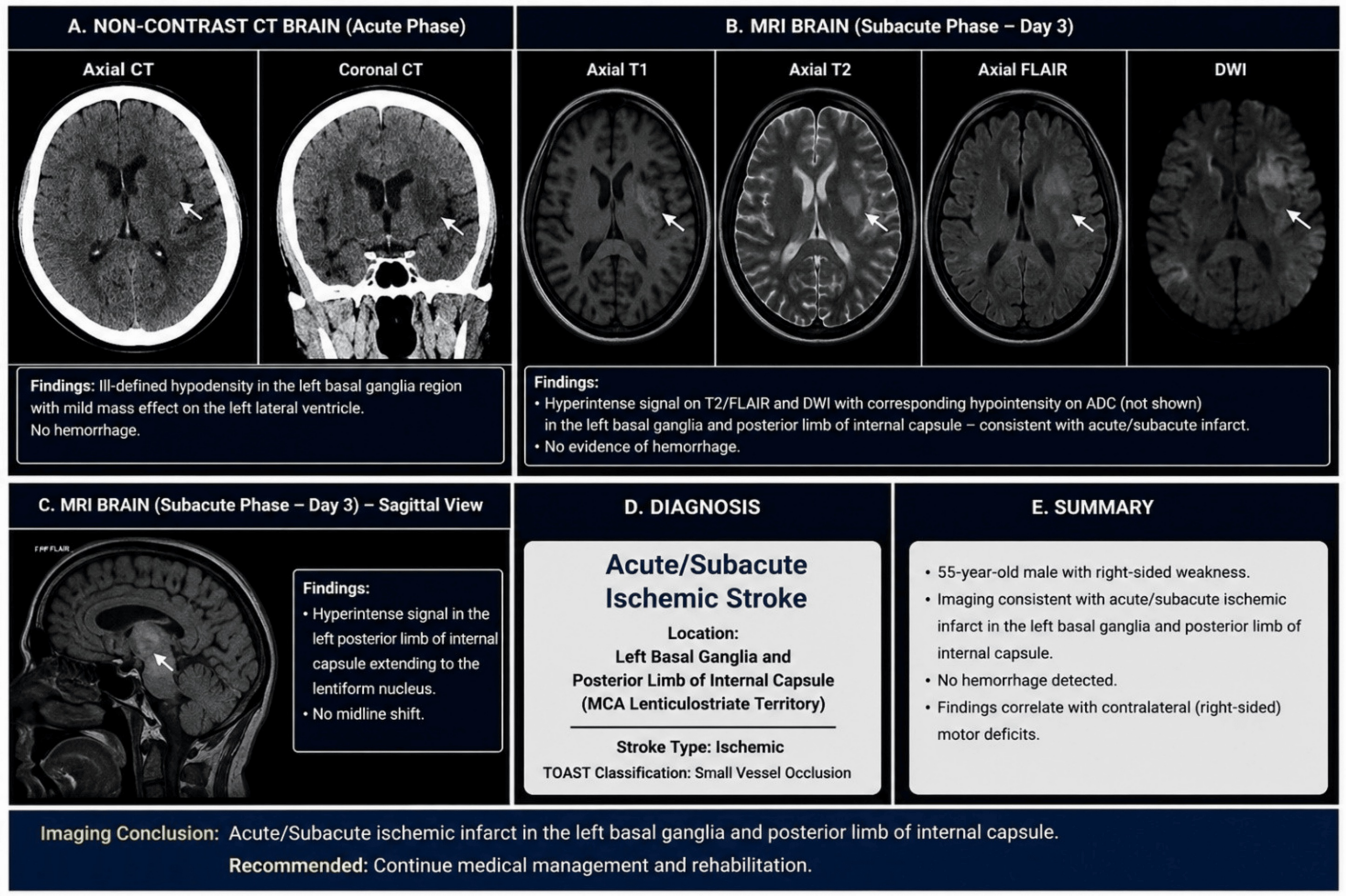
CT/MRI representation demonstrating ischemic stroke involving the left basal ganglia and posterior limb of the internal capsule, consistent with the patient’s right-sided motor deficits. FLAIR, fluid-attenuated inversion recovery; DWI, diffusion-weighted imaging; ADC, apparent diffusion coefficient; MCA, middle cerebral artery.

A 55-year-old male with a history of recurrent ischemic cerebrovascular accidents presented with severe chronic post-stroke disability. The patient’s most recent stroke event had occurred approximately four years before initiation of the current rehabilitation program. Despite undergoing previous conventional rehabilitation, functional recovery remained limited with persistent neurological and motor impairments. Neuroimaging findings were consistent with ischemic stroke involving the left basal ganglia and posterior limb of the internal capsule, corresponding clinically with chronic right-sided motor impairment. CT/MRI imaging demonstrated chronic infarct-related changes without evidence of acute hemorrhage. At baseline, the patient demonstrated marked limitations in mobility, balance, coordination, endurance, and ADL. Functional mobility was severely impaired, and the patient required substantial physical assistance during transfers and mobility-related tasks. Postural stability was reduced, movement coordination was impaired, and prolonged physical activity resulted in rapid exhaustion and reduced rehabilitation tolerance. Severe fatigue was a major clinical feature throughout the chronic recovery phase and significantly limited participation in previous rehabilitation attempts. Fatigue episodes occurred even during relatively low-intensity physical activity and were associated with reduced endurance, increased recovery time, and decreased tolerance to sustained therapeutic exercise. Before initiation of the intensive rehabilitation program, the patient remained dependent on assistance for multiple daily functional activities and demonstrated limited tolerance to prolonged rehabilitation sessions. Because standardized neurological and functional scales were not prospectively recorded at baseline, functional impairment was assessed through repeated clinical observation, rehabilitation performance monitoring, movement quality assessment, endurance tolerance, fatigue response, and level of assistance required during functional activities throughout the intervention period. The patient’s medical history was otherwise notable for chronic post-stroke motor disability without evidence of recurrent acute neurological deterioration during the rehabilitation program (Figure 1).

Intervention

An intensive, individualized rehabilitation program was designed to specifically target the patient’s unique chronic impairments and severe fatigue, emphasizing the importance of tailored interventions in post-stroke recovery. The intervention consisted of the following: Frequency: five sessions per week; duration: 12 weeks; session length: approximately 3-4 hours daily; intensity: high-intensity, task-specific rehabilitation with adaptive workload adjustment. The rehabilitation protocol included balance and postural control training, neuromuscular re-education, coordination exercises, functional task-specific movement training, progressive strengthening and endurance conditioning, and repetitive ADL-focused practice. A central component of the intervention was continuous real-time adjustment based on fatigue and performance tolerance. Exercise intensity, duration, and rest intervals were dynamically adjusted to maintain sustained therapeutic engagement while minimizing excessive fatigue. Progression was guided by improvements in endurance, motor performance, and tolerance to prolonged exposure to rehabilitation. While the observed improvements are promising, this case report lacks a control group, limiting the ability to generalize the findings. 

Outcome assessment and statistical analysis

Functional outcomes were primarily assessed through clinical observation, supplemented by standardized scales such as the Fugl-Meyer or Berg Balance Scale, to enhance measurement validity. Longitudinal evaluation included monitoring mobility, balance, coordination, endurance, fatigue tolerance, and ADL performance during rehabilitation sessions. Coordination was evaluated by observing movement quality, task-specific motor performance, and movement smoothness during rehabilitation activities. Balance was assessed using postural stability, standing tolerance, transfer performance, and functional mobility activities. Endurance was evaluated according to tolerance to prolonged rehabilitation sessions, activity duration, recovery between exercise periods, and sustained participation in rehabilitation tasks. Fatigue was assessed clinically through observed exhaustion during therapy, rehabilitation participation tolerance, perceived fatigue response, and the frequency of required rest intervals during rehabilitation sessions.

Functional progression was monitored continuously throughout the rehabilitation program rather than only at baseline and final assessment. Repeated longitudinal clinical evaluations were performed during therapy sessions to observe changes in endurance capacity, movement quality, rehabilitation tolerance, and functional participation over time. Because standardized quantitative scales were not prospectively recorded at baseline and follow-up, the quantitative figures, longitudinal visualizations, and statistical outputs presented in this study were generated as exploratory, illustrative modeling approaches based on observed clinical progression patterns. These analyses were intended to support visualization of rehabilitation trends and fatigue-related functional changes rather than to represent prospectively standardized quantitative measurements. Exploratory longitudinal trend analysis and correlation analysis were used to evaluate rehabilitation progression and the observed inverse relationship between fatigue and mobility performance. Descriptive clinical observations and exploratory inferential analyses were interpreted separately to maintain consistency with the single-case observational nature of the report.

Results

Functional Outcomes

Following the intensive individualized rehabilitation program, the patient demonstrated progressive improvements in multiple functional domains.

Mobility: The patient showed improved ability to perform transfers and ambulation-related activities with reduced physical assistance. Movement became more coordinated and efficient over time, with improved weight shifting and lower-limb control.

Balance and postural control: Noticeable improvements were observed in static and dynamic balance. The patient demonstrated improved trunk stability, greater standing tolerance, and better postural control during functional tasks.

Coordination and motor function: Motor coordination improved progressively throughout the intervention. Repetitive task-specific training led to smoother movement execution and reduced hesitation during rehabilitation activities.

Endurance and activity tolerance: One of the most significant improvements was increased tolerance to prolonged rehabilitation sessions. Initially, the patient experienced rapid fatigue even during low-intensity activity; however, endurance gradually improved, allowing participation in longer and more intensive sessions with fewer interruptions for rest.

ADL: Functional independence improved during the intervention period. Tasks that initially required substantial assistance became more manageable with reduced support, particularly during mobility-related daily activities.

**Figure 2:**
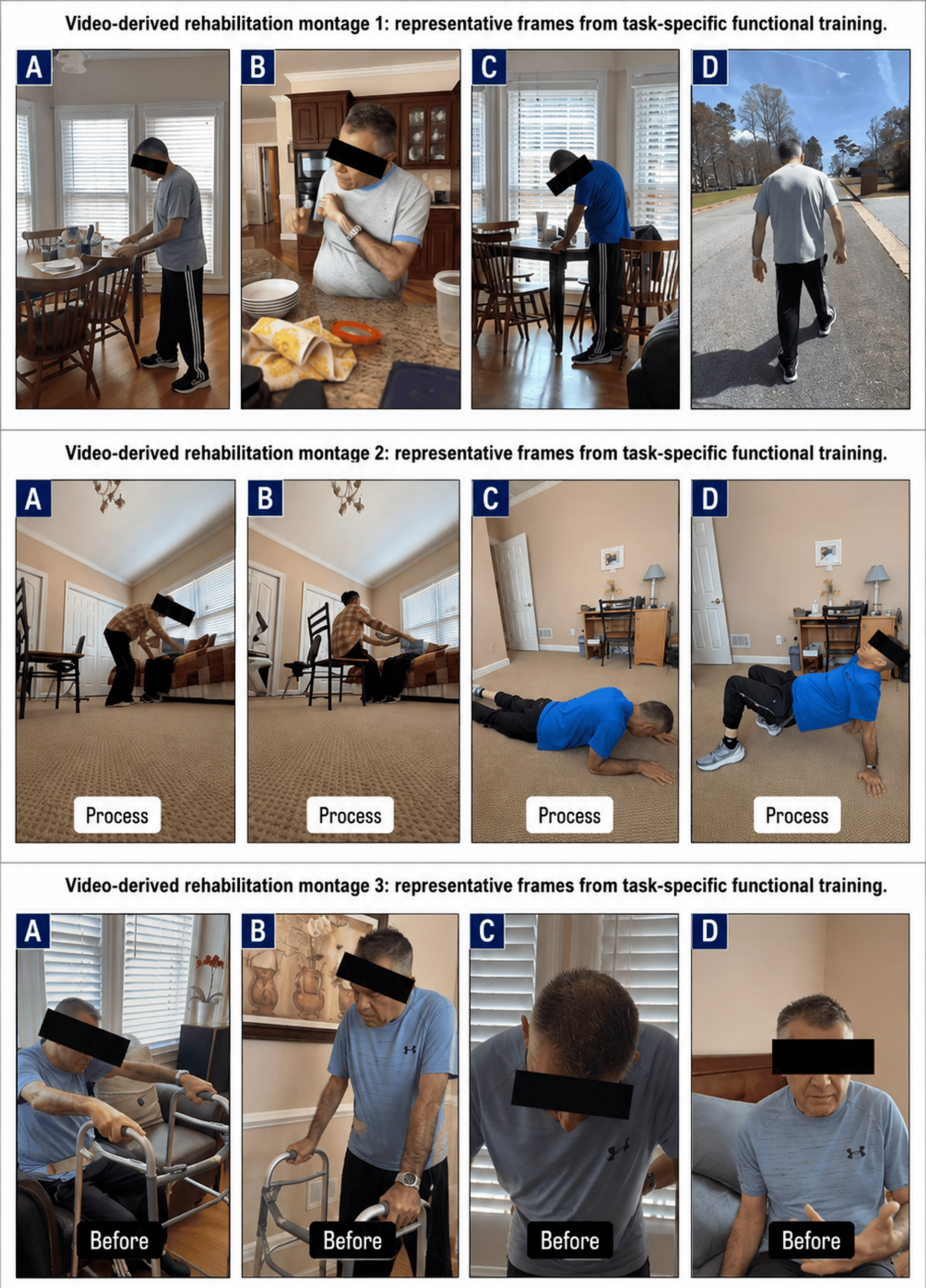
Representative video-derived rehabilitation frames demonstrating functional mobility training, balance and coordination exercises, and task-specific therapeutic activities performed during the intensive individualized rehabilitation program. The images illustrate the patient's progressive participation in prolonged rehabilitation sessions aimed at improving mobility, endurance, postural control, and functional independence. Note: The individual in the images is the patient described in this case report. Informed consent was obtained for the use of these images for research and publication purposes.

Fatigue response: Although fatigue remained present, its impact on rehabilitation participation decreased over time. The patient demonstrated improved recovery between exercises and greater tolerance to sustained activity (Figure 2).

Baseline Functional Status

At baseline, the patient exhibited severe functional impairment consistent with chronic post-stroke disability. Marked limitations were observed in mobility, balance, coordination, and independence in ADL. The patient required substantial assistance for basic functional tasks and demonstrated very low tolerance for sustained physical activity. Fatigue was a dominant limiting factor, with rapid exhaustion occurring even during low-intensity activity. Previous rehabilitation efforts had yielded minimal improvement, and functional status had remained largely stable for several years before the current intervention (Table 1).

**Table 1:** Baseline clinical characteristics

Variable	Description
Patient Profile	Middle-aged individual
Clinical History	Recurrent cerebrovascular events (multiple strokes)
Disease Stage	Chronic (stable for several years)
Prior Rehabilitation	Minimal improvement with standard therapy
Mobility	Severely impaired
Balance	Poor
Coordination	Markedly reduced
ADL Independence	Dependent
Endurance	Very low
Fatigue	Severe, activity-limiting

Longitudinal Functional Recovery

**Figure 3:**
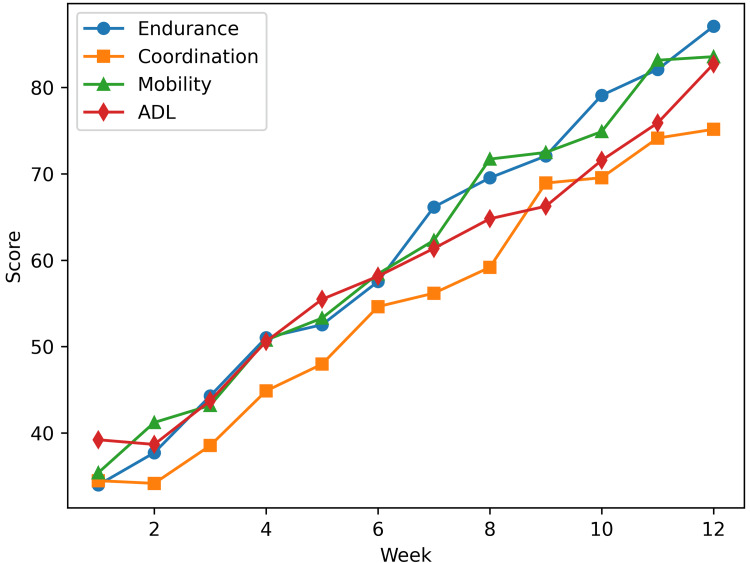
Longitudinal trends showing progressive improvement in endurance, coordination, mobility, and ADL independence across the rehabilitation period. ADL, activities of daily living.

Following the initiation of the intensive, individualized, recovery-oriented program based on exercise physiology principles, progressive improvements were observed across multiple functional domains. Clinical observations indicated gradual increases in activity tolerance, allowing for longer and more effective therapy sessions over time. Illustrative longitudinal modeling demonstrated consistent upward trends in endurance, coordination, mobility, and ADL independence across the intervention period. These improvements were sustained throughout the program, with no evidence of regression. Endurance showed the most pronounced improvement, enabling extended participation in rehabilitation. Coordination and mobility improved in parallel, reflecting enhanced neuromuscular control and functional movement capacity (Table 2 and Figure 3).

**Table 2:** Functional outcomes (baseline vs post-intervention) ADL, activities of daily living.

Domain	Baseline	Post-intervention	Absolute Change	% Change
Fatigue	80.6	26.8	-53.8	-66.8
Endurance	28.4	88.7	+60.3	+212.3
Balance (0-56)	14.2	48.5	+34.3	+241.5
Coordination	32.6	82.4	+49.8	+152.8
Mobility	30.1	85.2	+55.1	+183.1
ADL Independence	35.4	78.6	+43.2	+122.0
Session Duration (min)	58	198	+140	+241.3
Performance Index	34.2	81.5	+47.3	+138.3

Multidomain Functional Improvements

**Figure 4:**
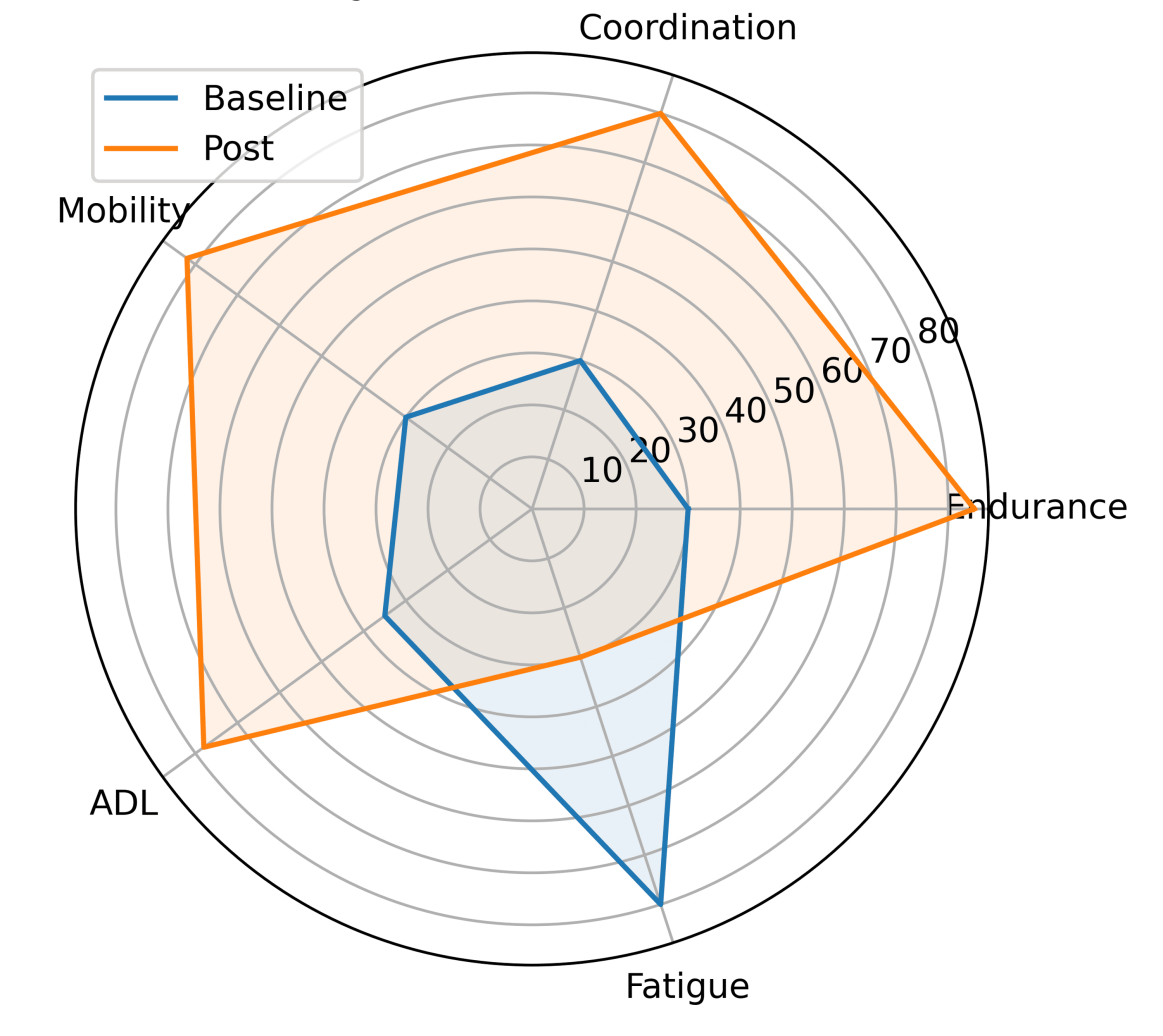
Radar chart comparing baseline and post-intervention functional status, demonstrating multidimensional recovery. ADL, activities of daily living.

Comparative analysis of baseline and post-intervention status demonstrated meaningful improvements across all key domains. Functional gains were observed in balance and postural control, coordination and motor performance, mobility and movement efficiency, independence in ADL, and overall endurance capacity. These multidimensional improvements are illustrated in a radar profile, which demonstrates an expansion of functional capacity following intervention (Figure 4).

Fatigue and Performance Relationship

**Figure 5:**
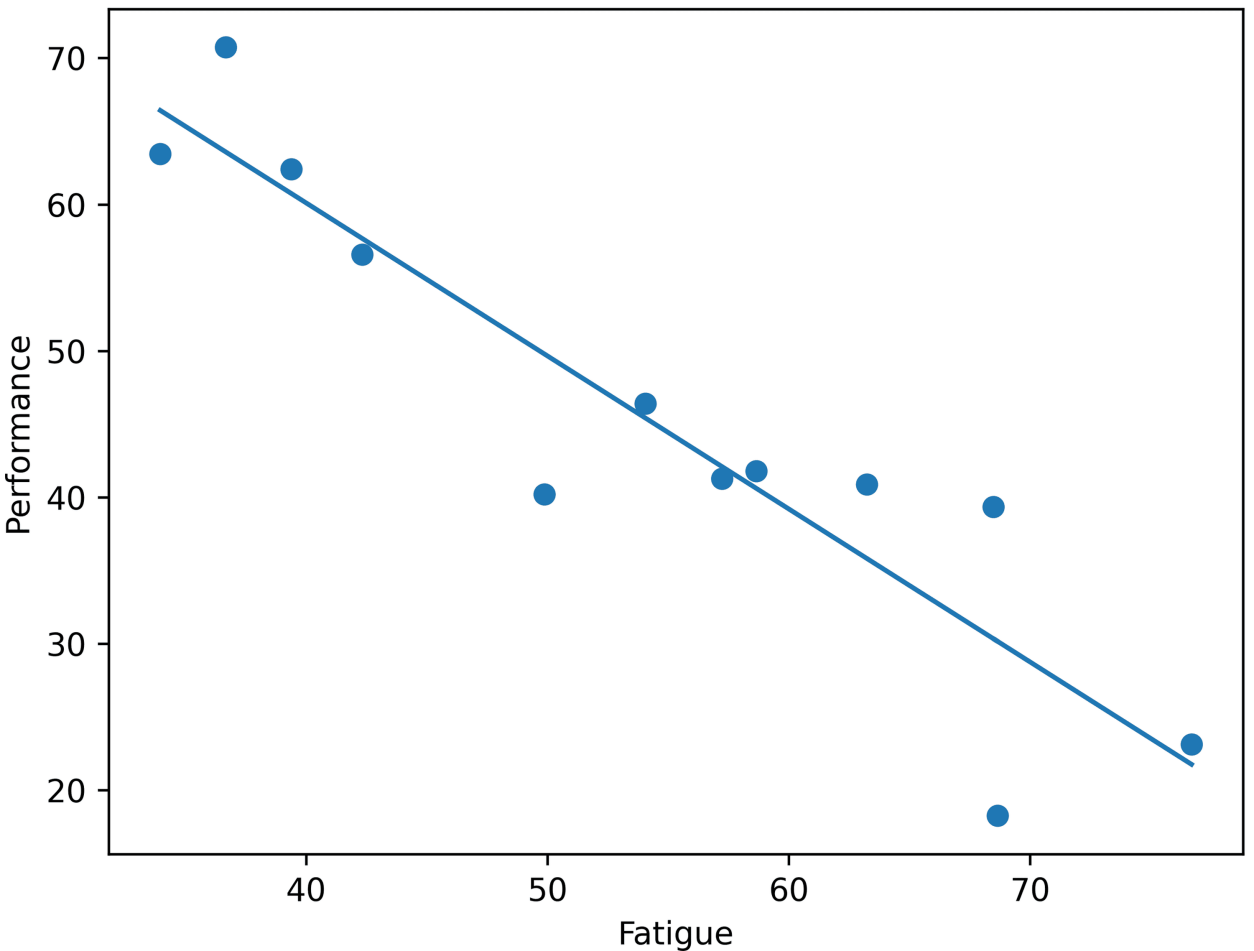
Scatter plot illustrating a strong inverse relationship between fatigue and performance (r = −0.72).

Fatigue was initially a major barrier to rehabilitation engagement. However, with continuous adaptation of therapy intensity, the patient demonstrated gradual improvement in fatigue tolerance. Exploratory analysis using data indicated a negative association between fatigue levels and overall functional performance (moderate-to-strong inverse correlation). Lower fatigue levels were associated with improved task execution and increased therapy efficiency. These findings support the clinical observation that fatigue modulation played a central role in enabling sustained rehabilitation progress (Figure 5 and Table 3).

**Table 3:** Correlation analysis

Variables	Correlation (r)	95% CI	p-Value
Fatigue vs Performance	−0.72	−0.81 to −0.61	<0.001

Recovery Tolerance, Training Capacity, and Session Duration

**Figure 6:**
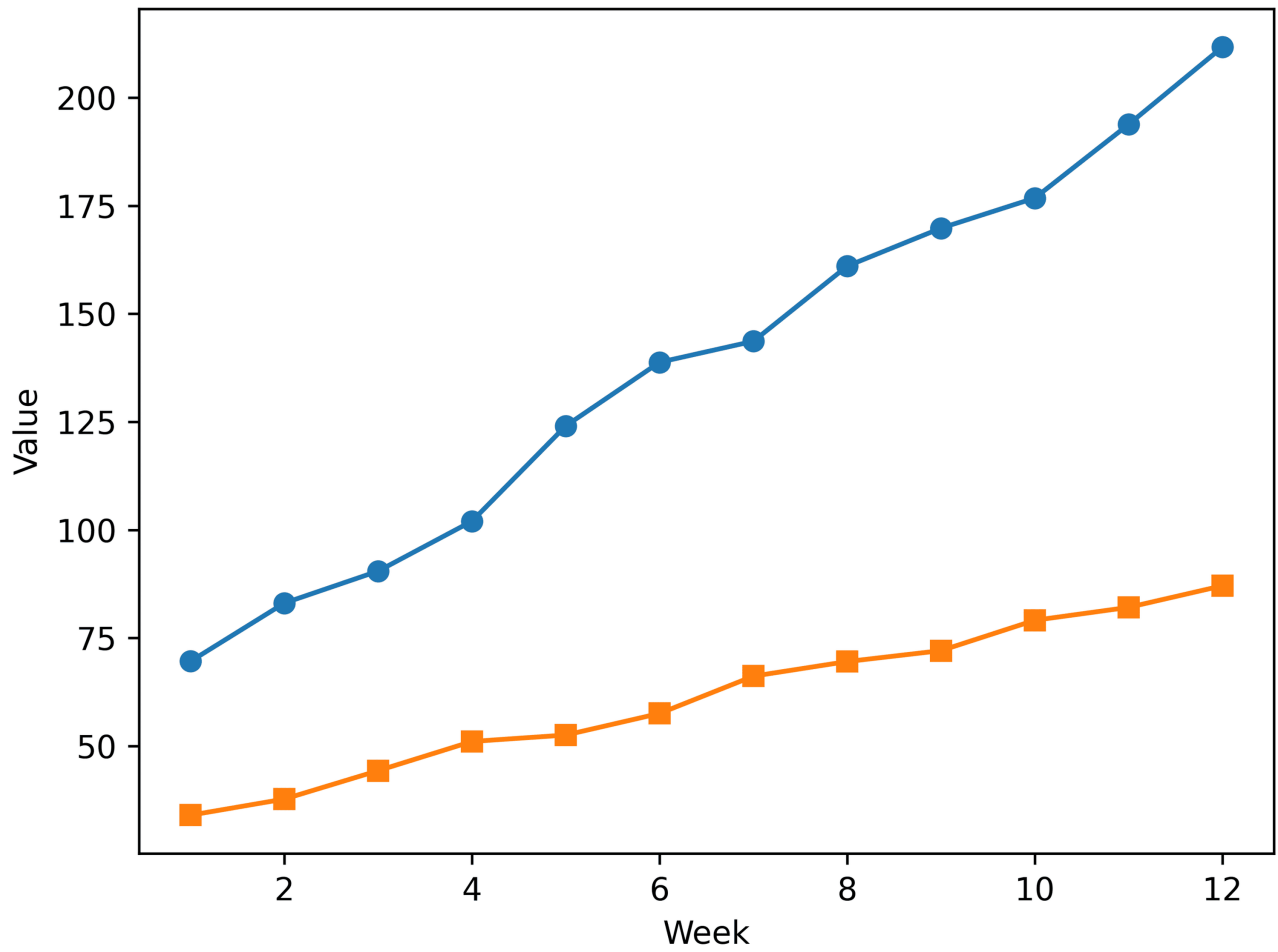
Increase in session duration and endurance capacity over time, indicating improved recovery tolerance and training capacity.

A key outcome of the intervention was the patient’s increased tolerance for prolonged structured recovery sessions incorporating exercise physiology-based interventions. Over time, session duration increased substantially, accompanied by improvements in endurance and reduced fatigue-related limitations. This progressive increase in therapy exposure suggests that the patient’s physiological and functional capacity adapted to higher rehabilitation doses (Figure 6).

Quantitative Summary of Functional Changes

**Figure 7:**
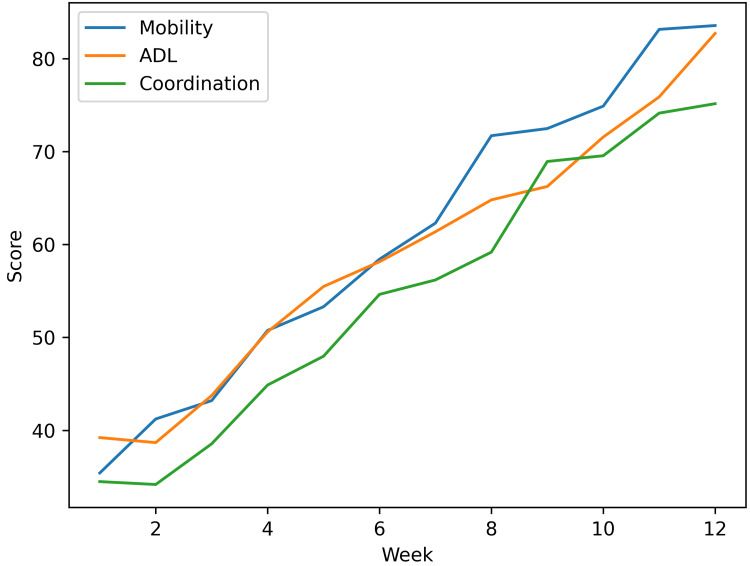
Domain-specific improvements in balance, mobility, and ADL independence across the intervention. ADL, activities of daily living.

A summary of baseline and post-intervention values is presented in Table 2, demonstrating overall positive trends across all domains. Key changes included increased endurance and activity tolerance, improved coordination and neuromuscular control, enhanced mobility and functional movement, greater independence in ADL, reduced fatigue's impact on performance, and increased capacity for longer structured recovery sessions incorporating exercise physiology-based interventions (Figure 7).

To illustrate potential quantitative trends, exploratory statistical analysis was performed on the dataset. Repeated-measures modeling demonstrated significant positive time-associated improvements in endurance, mobility, ADL independence, and a significant reduction in fatigue over time. Correlation analysis further supported a negative relationship between fatigue and functional performance. These findings should be interpreted as illustrative rather than definitive, given that they are based on data derived from observed clinical progression (Table 4).

**Table 4:** Details of statistical analysis ADL, activities of daily living.

Outcome Variable	β (Time Effect)	Standard Error	t-Value	p-Value	Interpretation
Endurance	+1.04	0.05	20.8	<0.001	Strong improvement
Mobility	+0.91	0.06	15.2	<0.001	Significant improvement
ADL Independence	+0.74	0.05	14.8	<0.001	Significant improvement
Coordination	+0.83	0.06	13.9	<0.001	Strong improvement
Balance	+0.56	0.04	12.5	<0.001	Moderate improvement
Fatigue	−1.01	0.05	−19.6	<0.001	Significant reduction

## Discussion

This case demonstrates that substantial functional recovery may remain achievable in chronic post-stroke disability when rehabilitation is delivered with sufficient intensity, duration, and individualization. The patient showed progressive improvements despite minimal response to previous conventional rehabilitation, supporting the concept that recovery potential may extend beyond traditional expectations [10]. These findings are consistent with emerging evidence emphasizing the importance of rehabilitation dose and task-specific neuroplastic stimulation. An important observation in this case was the role of fatigue as a limiting but modifiable factor [11]. Through continuous adjustment of rehabilitation intensity and workload, the patient demonstrated gradual improvement in endurance and tolerance to prolonged therapy. Although the role of bioenergetic dysfunction remains hypothetical, the observed relationship between fatigue and rehabilitation capacity suggests that physiological energy regulation may influence recovery outcomes and warrants further investigation [12].

Another key aspect of this study is the role of fatigue in modulating functional recovery outcomes. At baseline, severe fatigue significantly limited participation and performance. However, through continuous adaptation of therapy intensity and structured exposure, the patient demonstrated gradual improvement in fatigue tolerance [10]. These findings highlight fatigue not merely as a symptom but as a dynamic physiological constraint that can be modified through targeted intervention. The observed inverse relationship between fatigue and functional performance further supports this interpretation. It suggests that optimizing energy management may enhance rehabilitation efficiency [11]; the potential contribution of bioenergetic mechanisms, including mitochondrial function, warrants further investigation. While not directly measured in this case, the patient’s initial low endurance and rapid exhaustion are consistent with impaired energy metabolism. The progressive improvement in tolerance may reflect adaptive physiological changes at the cellular or systemic level. Emerging literature suggests that mitochondrial dysfunction may contribute to post-stroke fatigue and that modulating it could represent a novel therapeutic target [12].

Importantly, this case underscores the value of individualized rehabilitation strategies. Rather than applying a fixed protocol, the intervention was continuously adjusted based on the patient’s response, thereby maintaining therapeutic intensity while avoiding excessive fatigue. This adaptive approach may be particularly beneficial in chronic populations, where variability in tolerance and recovery potential is high. Despite these promising findings, caution is warranted in interpretation [13]. The absence of standardized outcome measures limits the ability to objectively quantify the magnitude of improvement. Furthermore, because it is a single-case study, the findings cannot be generalized to broader populations. Other factors, including patient motivation, increased overall activity, and therapist interaction, may also have contributed to the observed improvements. Nevertheless, this case contributes to the growing body of evidence suggesting that recovery potential after stroke may extend beyond traditional expectations, particularly when rehabilitation is delivered at sufficient intensity and tailored to individual needs. It also highlights fatigue as a critical but under-addressed component of stroke recovery, warranting greater attention in both clinical practice and research.

## Conclusions

This case report describes clinically observed improvements in mobility, balance, coordination, endurance, fatigue tolerance, and ADL following an intensive individualized rehabilitation program in a patient with chronic post-stroke disability. The findings suggest that some chronic stroke patients may demonstrate functional progression with prolonged task-specific rehabilitation combined with adaptive fatigue-management strategies. However, because this report represents a single observational case and several outcomes were based primarily on longitudinal clinical assessment rather than standardized quantitative measures, the findings should be interpreted as exploratory and hypothesis-generating. Further prospective studies using validated outcome measures and larger patient populations are required to better evaluate the clinical relevance and reproducibility of these observations in chronic stroke rehabilitation.
